# Reversal of Peripheral Anterior Synechiae After Trabeculectomy With Argon Laser Synechiolysis

**DOI:** 10.7759/cureus.59668

**Published:** 2024-05-05

**Authors:** Inderpreet Kaur, Norshamsiah Md Din, Jemaima Che Hamzah, Rupini Yogesvaran

**Affiliations:** 1 Department of Ophthalmology, Hospital Canselor Tuanku Muhriz UKM (HCTM), Selangor, MYS; 2 Department of Ophthalmology, Universiti Kebangsaan Malaysia Medical Centre, Kuala Lumpur, MYS; 3 Department of Ophthalmology, Faculty of Medicine, Universiti Kebangsaan Malaysia, Kuala Lumpur, MYS; 4 Department of Ophthalmology, Hospital Tengku Ampuan Rahimah, Klang, MYS

**Keywords:** filtering glaucoma surgery, glaucoma surgeries, secondary glaucoma, lasers retina and glaucoma, steroid induced glaucoma

## Abstract

We report a case of successful argon laser synechiolysis as a non-invasive alternative for peripheral anterior synechiae release after trabeculectomy in a young patient with steroid-induced ocular hypertension. Steroid-induced ocular hypertension is a known complication of vernal keratoconjunctivitis due to prolonged treatment with steroids. In refractive conditions, augmented trabeculectomy becomes the surgery of choice in these patients.^ ^In this article, we report successful treatment of iris tissue plugging the internal ostium with an argon laser and reinstatement of aqueous flow.

## Introduction

Vernal keratoconjunctivitis (VKC) is a chronic disease that may require a prolonged duration of topical steroids to downregulate the disease activity in moderate severity [1]. Topical ophthalmic corticosteroids usually lead to a rise in intraocular pressure (IOP) after three to six weeks of continuous administration, although a rise may be observed within one week of initiating treatment. Steroid use in VKC is often prolonged and may require high potency usage. This may lead to complications of secondary high intraocular pressure which may require surgical intervention in refractory cases [1]. Trabeculectomy is one of the options for glaucoma surgery where it creates an alternate route for aqueous outflow via an ostium. Despite the best efforts, post-operative complications may occur which include bleb leakage, hypotony with maculopathy, choroidal effusions, overfiltrating or encapsulated bleb, endophthalmitis, and malignant glaucoma. One of the post-operative complications also includes obstruction to flow at the sclerotomy site by peripheral anterior synechiae (PAS) leading to secondary high intraocular pressure and bleb failure. Argon laser has been used indirectly to shrink the iris tissue away from the point of attachment to relieve the obstructed ostia. We report a case of successful reinstatement of aqueous outflow post-treatment of iris tissue plugging the internal ostium via Argon laser as a non-invasive alternative in a young patient with steroid-induced ocular hypertension who has undergone trabeculectomy.

## Case presentation

A 15-year-old male with underlying vernal keratoconjunctivitis developed both eye steroid-induced ocular hypertension a month after commencing steroid treatment. He received regular topical dexamethasone 1% eye drops every 4 hours in both eyes and due to the severity of VKC complicated with shield ulcers, supratarsal triamcinolone at a dose of 16mg in the right eye (RE) and 20mg in the left eye (LE) was administered at the third week of treatment. He developed secondary steroid-induced ocular hypertension within a month of steroid commencement and was started on topical antiglaucoma G Latanoprost at night, G Brinzolamide (Azopt) thrice a day, and G Timocomod twice a day. However, due to his underlying VKC and worsening ocular surface disease, an oral acetazolamide dose of 250mg BD was prescribed. Topical steroid medications were withheld and the steroid-sparing agent, G cyclosporine, was continued upon further development of steroid-induced ocular hypertension. His preoperative vision was 6/12 with non-injected conjunctiva at 1 month of VKC treatment preoperatively and improving upper tarsal macropapilae superiorly. Fundus examination was unremarkable with no visual field defect and a cup-disc ratio (CDR) of 0.4 bilaterally. He underwent LE augmented trabeculectomy with Mitomycin C (MMC) 0.02% for uncontrolled IOP with maximum tolerated medical therapy which ranged at 28-30 mmHg on treatment. Postoperatively, his current LE IOP was 10mmHg without medication, with a vision of 6/12, a CDR of 0.4, and a healthy neuroretinal rim. The shield ulcer was treated, however, he developed a superior corneal scar as a complication.

Three months later, he underwent RE augmented trabeculectomy with MMC C 0.02% which was complicated by hypotony and shallow anterior chamber (AC) due to a limbal wound leak. Vision, which was 6/12 preoperatively, dropped to 6/36 on postoperative day 1. It was initially treated conservatively with a large diameter (18mm) bandage contact lens. Conjunctival resuturing was done on day 7 for a persistent leak, hypotony, and hypotony maculopathy. His condition improved with improvement in vision to 6/12, IOP stabilized to 12mmHg, the AC deepened, and hypotony complications resolved.

However, at six weeks postoperatively, his vision worsened to 6/36 pin hole 6/12 due to dry cornea surface and cornea bedewing, and his IOP increased to 22mmHg. A full dilated eye examination showed unremarkable posterior segment findings. A diagnosis of impending bleb failure was made based on the clinical features of progressive increase in IOP accompanied by flattening of the bleb. The releasable sutures were removed and his IOP came down to 14mmHg, however, the bleb morphology did not improve.

Bleb needling under a microscope with 5-fluorouracil and 30 Gauge needle was performed under local anaesthesia in a procedure room with aseptic technique and periocular povidone was used to clean the eye. However, the procedure was abandoned asthe patient complained of sudden eye pain, headache, and nausea mid-procedure. The rechecked IOP was recorded as 44mmHg. As the AC was deep, malignant glaucoma was ruled out and posterior segment examination did not show any suprachoroidal effusion or bleed.

**Figure 1:**
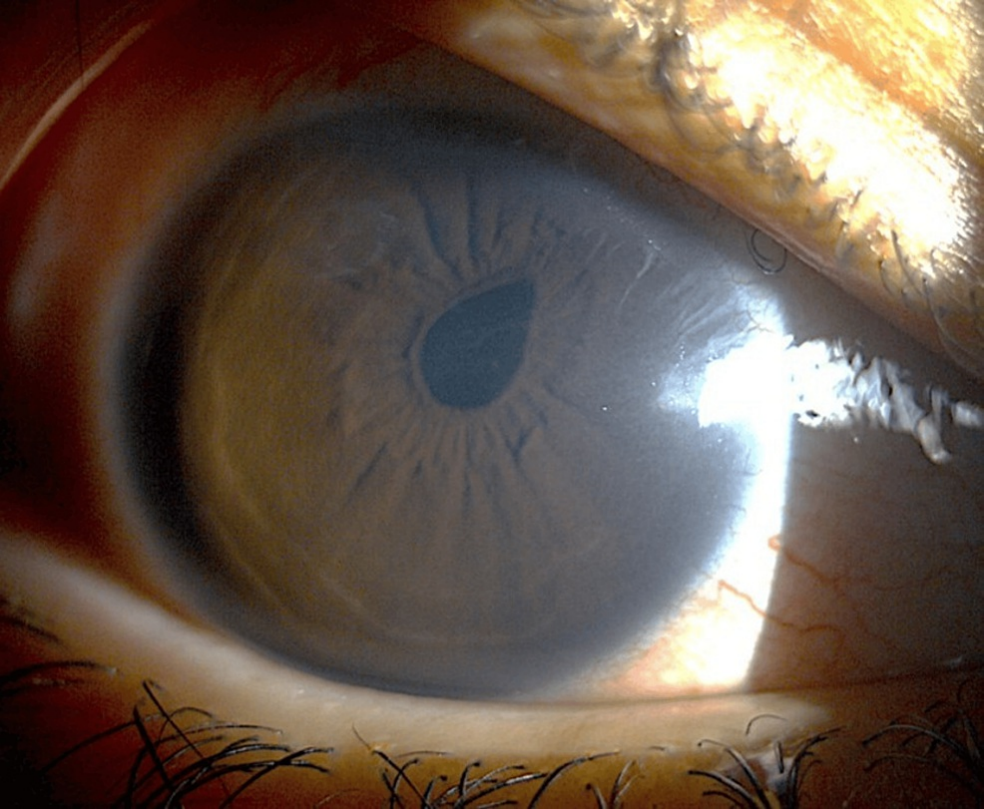
Peaked pupil with peripheral anterior synechiae at the superonasal site

**Figure 2:**
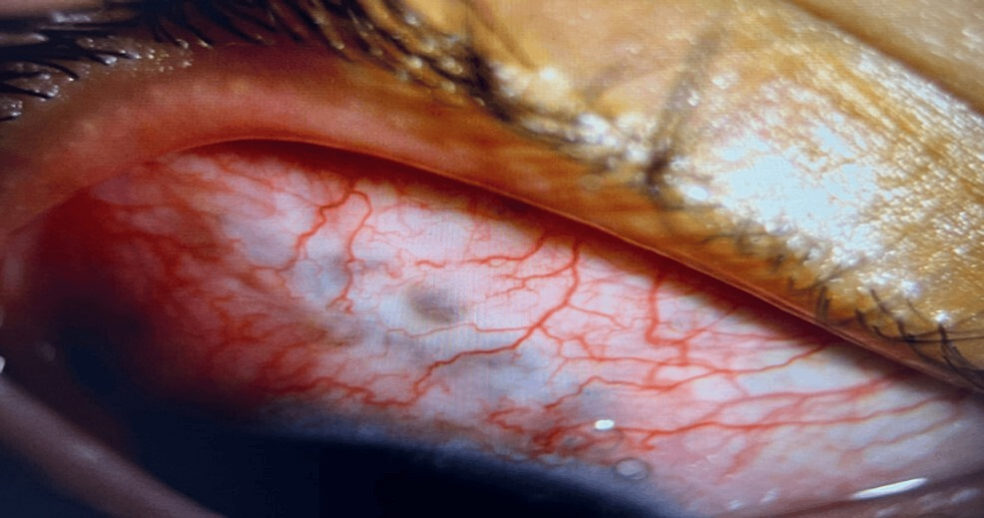
Bleb is thin-walled, hyperemic and non-cystic

The next day after needling, his IOP was unrecordable due to hypotony, the AC was shallow, and there was a wound leak from the needle puncture site which was not visualized immediately post-procedure. The puncture site was sutured and his condition stabilized after 10 days. His vision returned to 6/12, the AC deepened, and the IOP dropped to 6mmHg, without any hypotony complications. However, the pupil peaked towards the trabeculectomy site and pigments were seen at the subconjunctival area under the bleb (Figure 1). The bleb morphology was thin-walled, non-cystic, and hyperemic with iris pigments (Figure 2).

**Figure 3:**
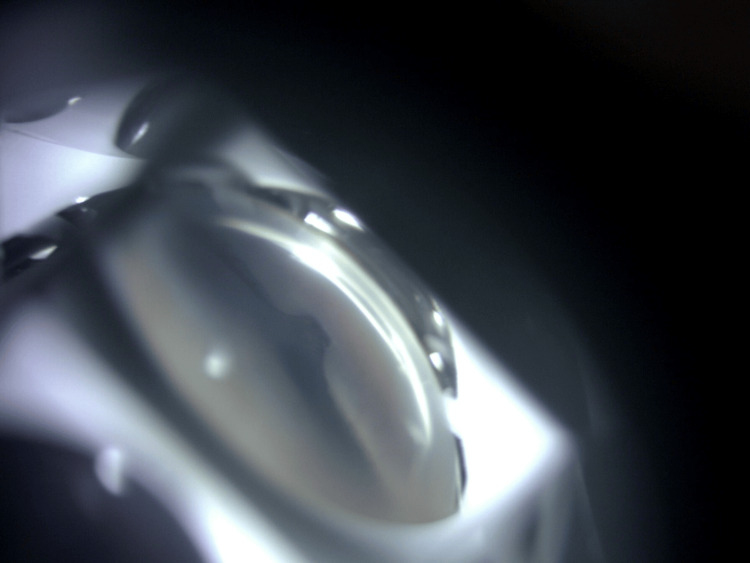
Gonioscopy showing a tuft of iris tissue plugging the internal ostium

**Figure 4:**
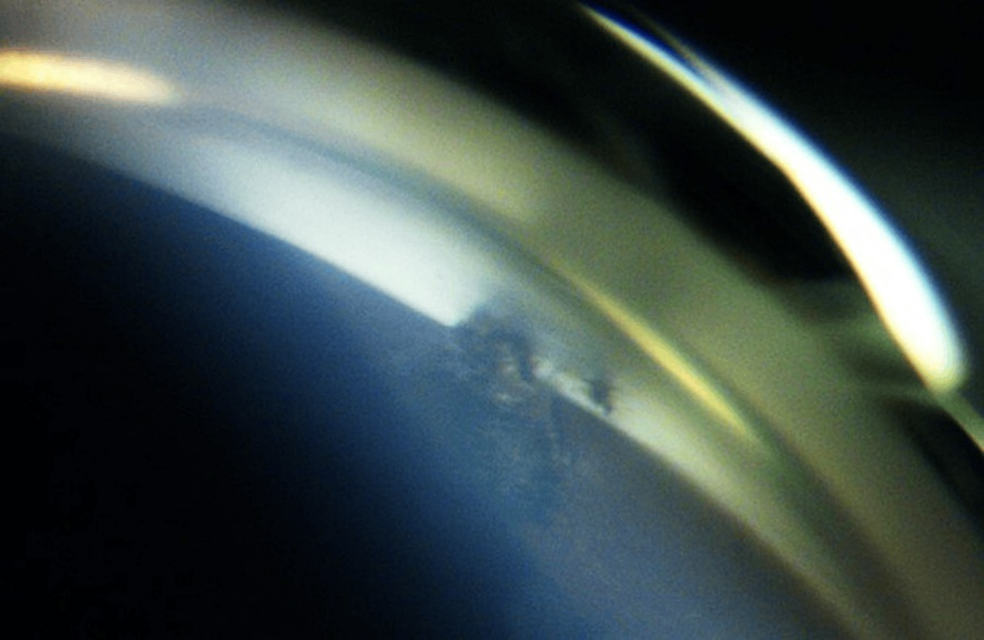
Broken peripheral anterior synechiae during argon laser goniosynechiolysis at 12 weeks postoperatively

**Figure 5:**
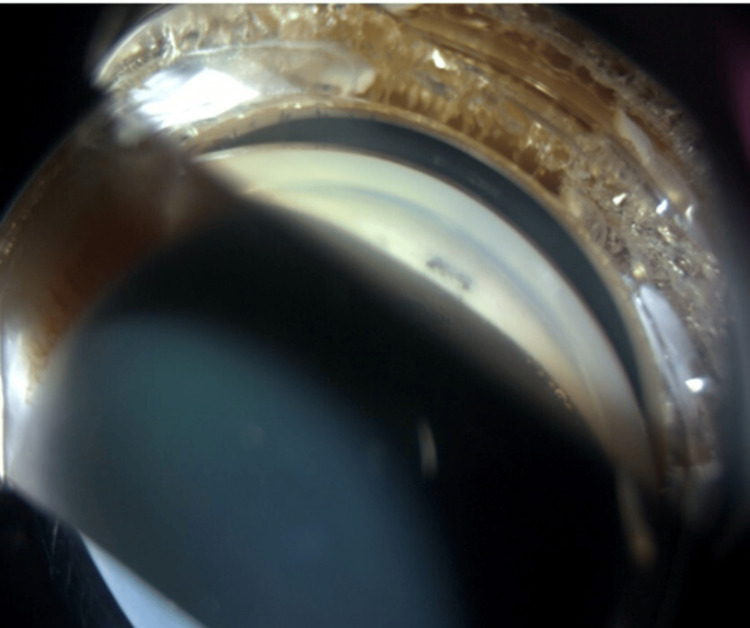
Post-argon laser goniosynechiolysis showing broken peripheral anterior synechiae with patent external ostium

Gonioscopy revealed a tuft of iris tissue was plugging the internal ostium (Figure 3). We attempted argon laser synechiolysis using a Magna view gonio lens with a spot size of 50 $\mu$m, 0.2 second, and power of 500-800mW. We managed to successfully release the iris tissue from the internal ostium (Figure 4-5).

The IOP post-procedure was 10mmHg in the RE. Subsequently, he has required bleb needling with subconjunctival 5-fluorouracil injection twice. At 6 months post trabeculectomy, the bleb is well-functioning and the IOP ranged between 08-13 mmHg without any IOP-lowering medication. His optic nerve is also healthy without any glaucomatous optic neuropathy.

## Discussion

Topical ophthalmic corticosteroids usually lead to a rise in IOP after three to six weeks of continuous administration. However, a rise in IOP may be observed within one week of initiating treatment [2]. Intervention and early treatment are required in patients with ocular hypertension as 9.5% of these patients eventually develop glaucomatous optic neuropathy and associated visual field loss versus 4.4% in those receiving IOP-lowering medication 5 years later [2].

In steroid-induced ocular hypertension and steroid-induced glaucoma, augmented trabeculectomy and tube shunts become the surgery of choice in patients in refractive conditions [2,3]. Reduction of IOP and maintaining it within the target IOP range is the main aim of trabeculectomy. However, to achieve this desired result, meticulous post-operative care is required to achieve a long-term functioning trabeculectomy bleb. Early postoperative IOP abnormalities can be varied, as demonstrated in our case. Early hypotony commonly occurs due to a wound leak or overfiltration, while high IOP could be due to tight closure of the scleral flap, malignant glaucoma, suprachoroidal hemorrhage, or obstruction of the sclerostomy site by iris, vitreous, ciliary body, a large blood clot, fibrin or viscoelastic [4]. In our case, an early hypotony due to the wound leak led to a shallow anterior chamber. This probably caused recurrent iridocorneal contact and eventually contributed to an occlusion of the sclerostomy site by some iris tissue. This was further exacerbated by performing bleb needling in a pharmacologically dilated eye leading to iris incarceration. We highlight the importance of gonioscopy in eyes with high IOP post-trabeculectomy to correctly identify the site of the obstacle as gonioscopy is a basic clinical tool that is often overlooked but useful in primary settings.

The strategy in a complication like this is to remove the obstruction (PAS) and restore filtration through the ostium. In very early cases, blockage may be successfully released by topical miotic eye drops [5]. Treatment with argon laser has been reported to be successful in reversing obstructed ostium by pigmented tissue in early or late phases [6]. Argon laser can be used indirectly at the iris adjacent to the synechiae to shrink the iris tissue away from the point of attachment. This is frequently achieved using a low-powered, long-duration, and larger spot-size laser beam similar to iridoplasty settings [4]. Alternatively, as what we had attempted, direct laser shots can be aimed at the synechiae using a smaller spot size (50-100 $\mu$m), higher power (up to 1000mW), and a shorter exposure time (0.1-0.2s) [4,7]. Other methods of laser includes using Nd:YAG laser directly, but with less success on pigmented tissue and in firmly adhering synechiae [4,8]. Unfortunately, if medical and laser therapy were futile, synechiolysis needs to be attempted manually via surgical intervention using viscoelastic needles or microforceps.

## Conclusions

Argon laser synechiolysis is advantageous in comparison to conventional surgical methods for several reasons. Laser therapy can be administered in a clinic setting, requiring only topical anesthesia, and the risks of surgery, such as bleeding and injury to the adjacent structures including the lens, is lower with laser therapy. This method can also be repeated safely. In conclusion, argon laser synechiolysis is a suitable alternative for treating PAS occluding the internal ostium as it is non-invasive and superior to surgical revisions/synechiolysis in terms of complications. It can successfully restore aqueous flow through the sclerostomy once the obstruction has been removed, maintaining a desirable IOP.
